# Supplementary material for: Microbiome analysis of 940 lung cancers in never-smokers reveals lack of clinically relevant associations
Source: Nat Commun. 2025 Dec 12;17:192. doi: 10.1038/s41467-025-66780-y (PMC12780107; doi:10.1038/s41467-025-66780-y)
Supplement: Supplementary file 1 — Supplementary Information [file 41467_2025_66780_MOESM1_ESM.pdf]

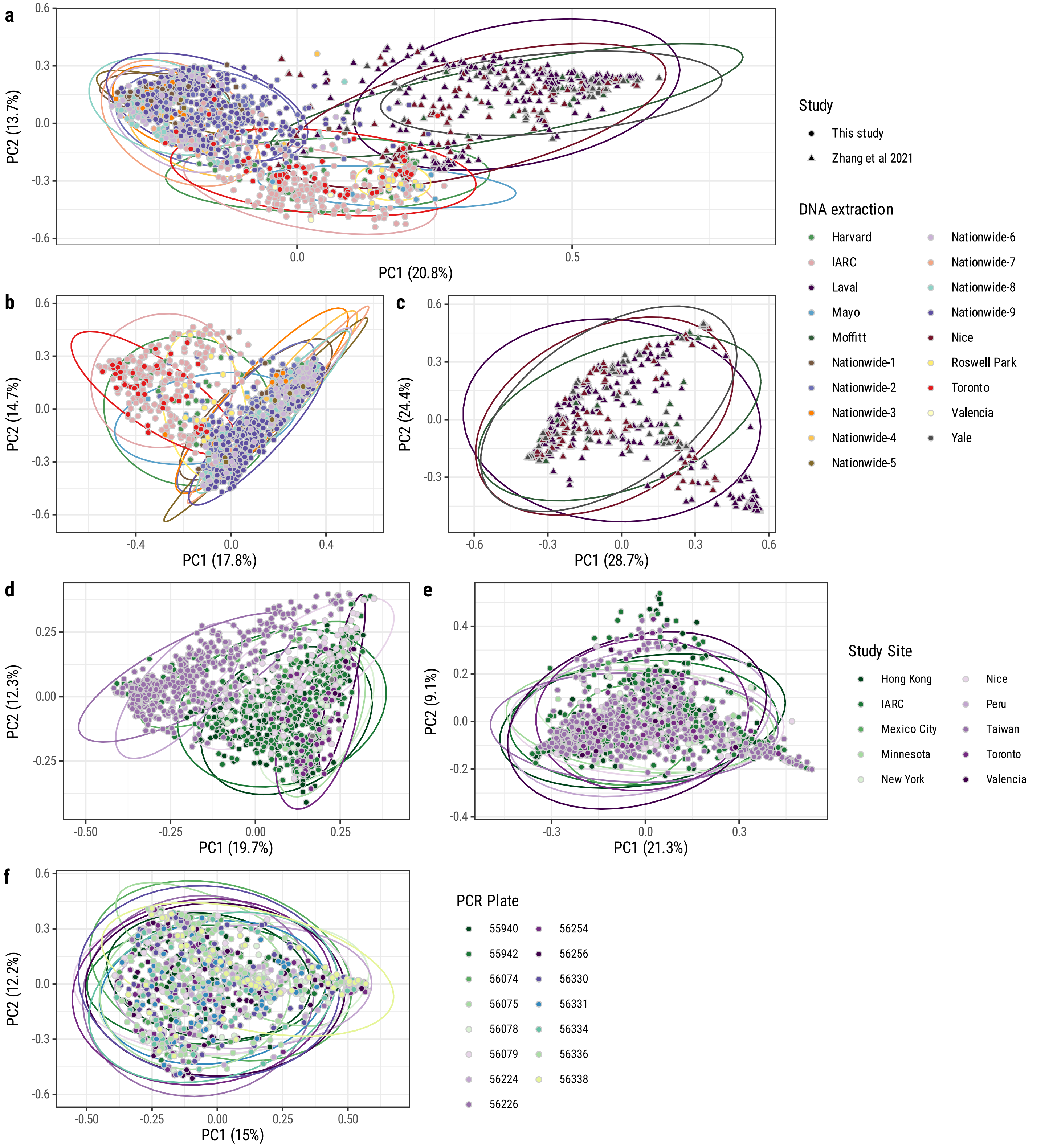

Supplementary Figure 1: a) Principal Coordinates Analysis (PCoA) of WGS data before batch correction and decontamination, and b) WGS samples from this study and c) WGS samples from Zhang et al. 2021 after batch correction and decontamination. d) PCoA of RNA-seq data before and e) after batch correction and decontamination. f) PCoA of 16S data after decontamination alone.

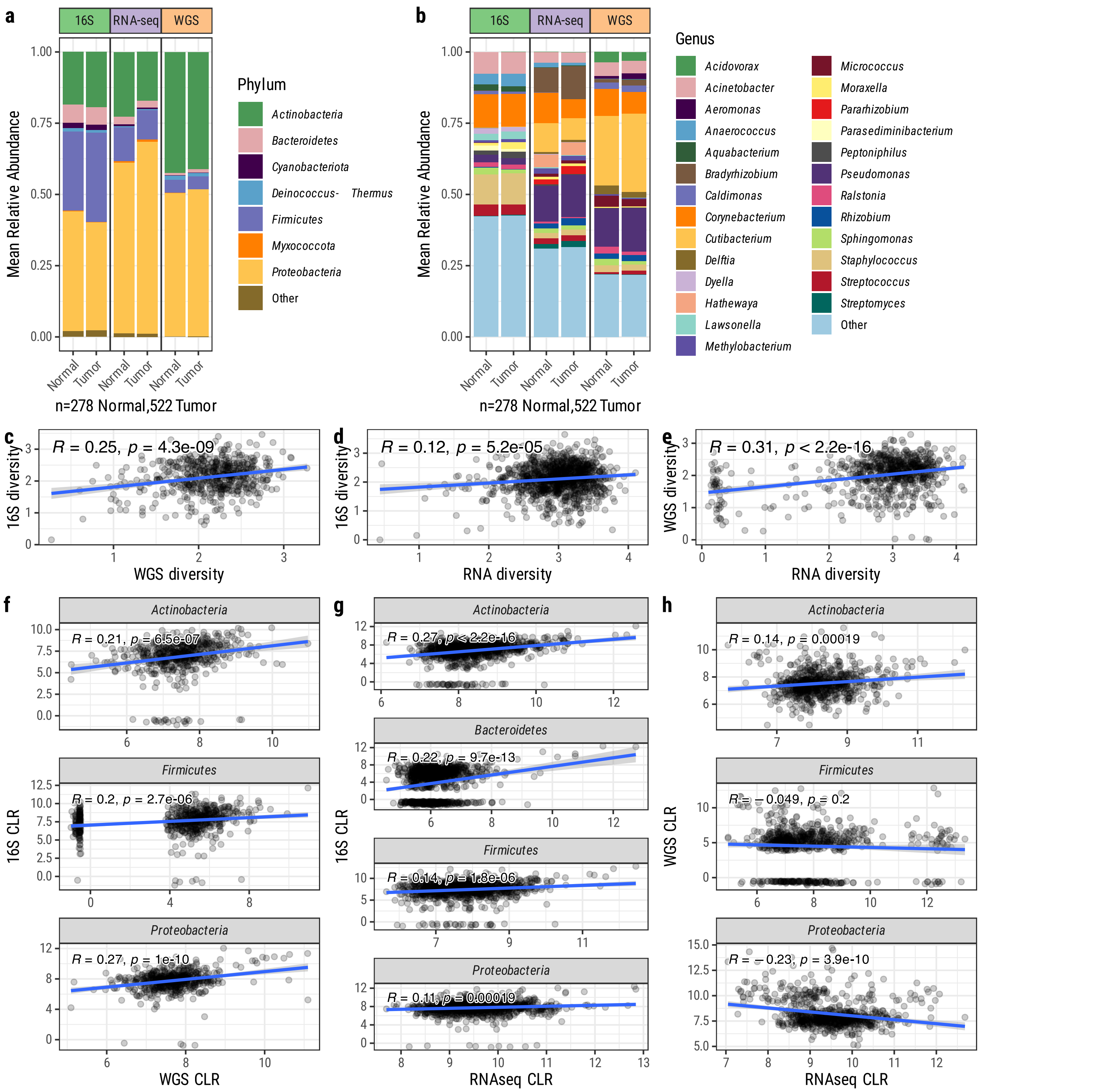

Supplementary Figure 2: Microbiome composition and intra-class correlations prior to contaminant filtering and batch correction. a) Mean phylum-level and b) genus-level relative abundances by sequencing platform and tumor-normal status, including only samples which were sequenced across all three sequencing modalities. c-e) Intra-class correlations of genus-level Shannon alpha diversity comparing samples across 16S and WGS (*n*=546), 16S and RNA-seq (*n*=1,075), WGS and RNA-seq (*n*=694) datasets. f-h) Intra-class correlation of phylum-level center log ratio (CLR) abundances comparing samples across 16S and WGS (*n*=553), 16S and RNA-seq (*n*=1,075), WGS and RNA-seq (*n*=701) datasets.

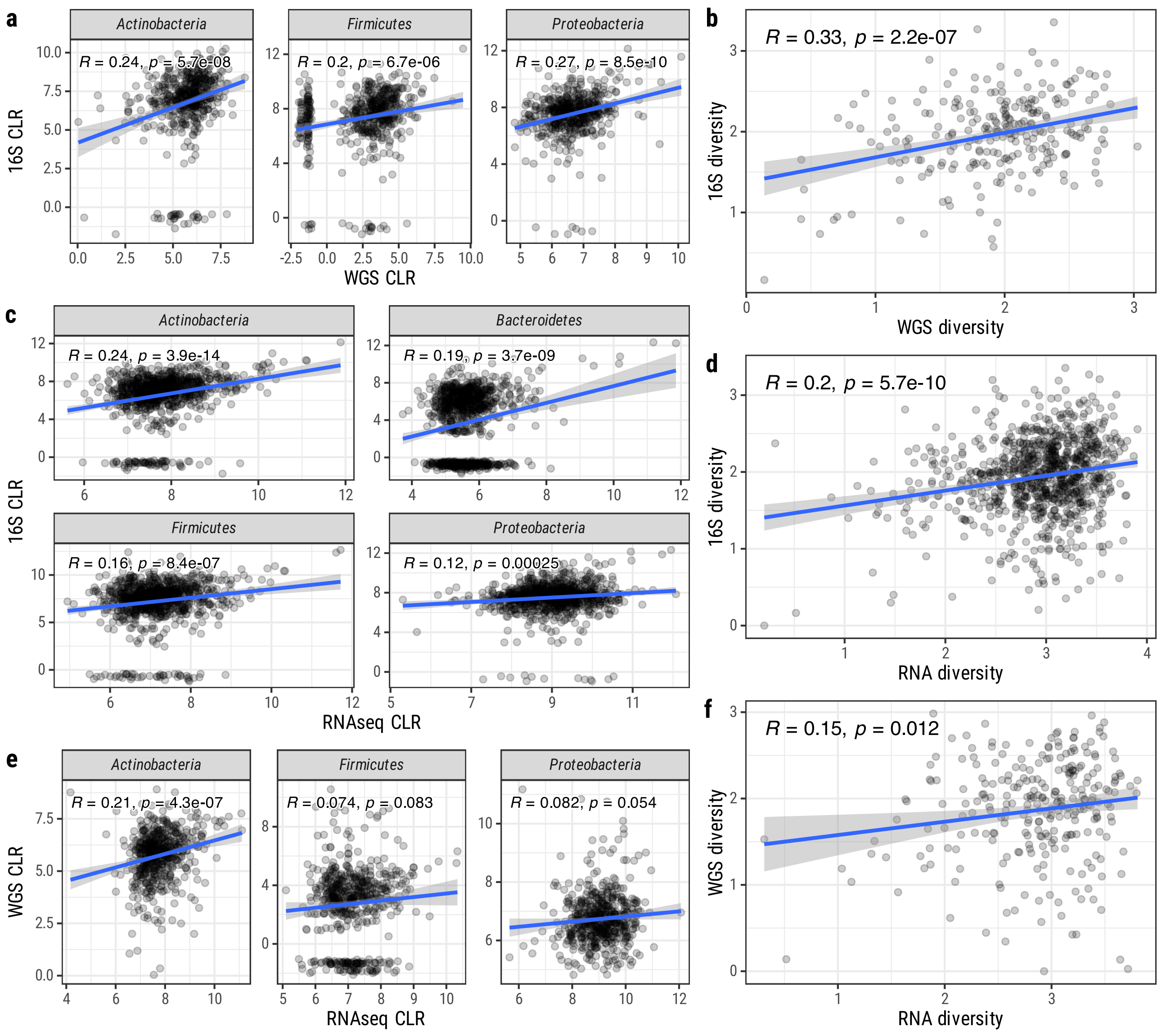

Supplementary Figure 3: Microbiome intra-class correlations after contaminant filtering and batch correction. a) Phylum-level abundance and b) genus-level Shannon alpha diversity correlations within samples sequenced via WGS and 16S (n=514). c) Phylum-level abundance and d) genus-level Shannon alpha diversity correlations within samples sequenced via 16S and RNA-seq (n=944). e) Phylum-level abundance and f) genus-level Shannon alpha diversity correlations within samples sequenced via WGS and RNA-seq (n=557). A 250 reads minimum cutoff was applied to all samples at the phylum level for relative abundance comparisons, and at the genus-level for diversity comparisons. Only phyla with at least 50% prevalence in all datasets were included for correlation. Diversity correlations were calculated after rarefying to 250 reads in all samples. *R*=Pearson correlation coefficient.

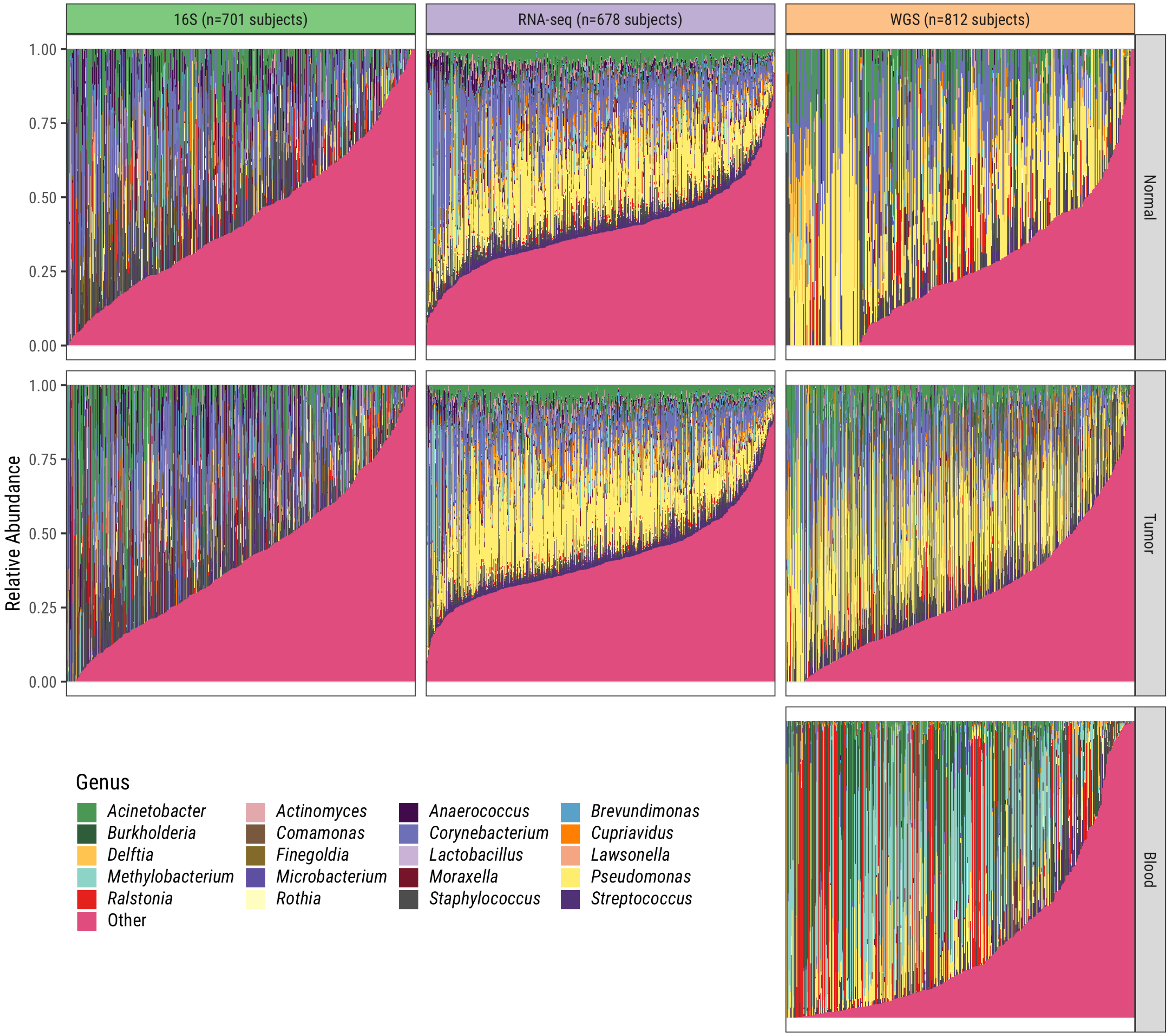

Supplementary Figure 4: Overview of the genus-level relative abundances for all samples in this dataset. The twenty most common bacterial genera are shown, and samples are ordered along the x-axis by the relative abundance of "Other" genera.

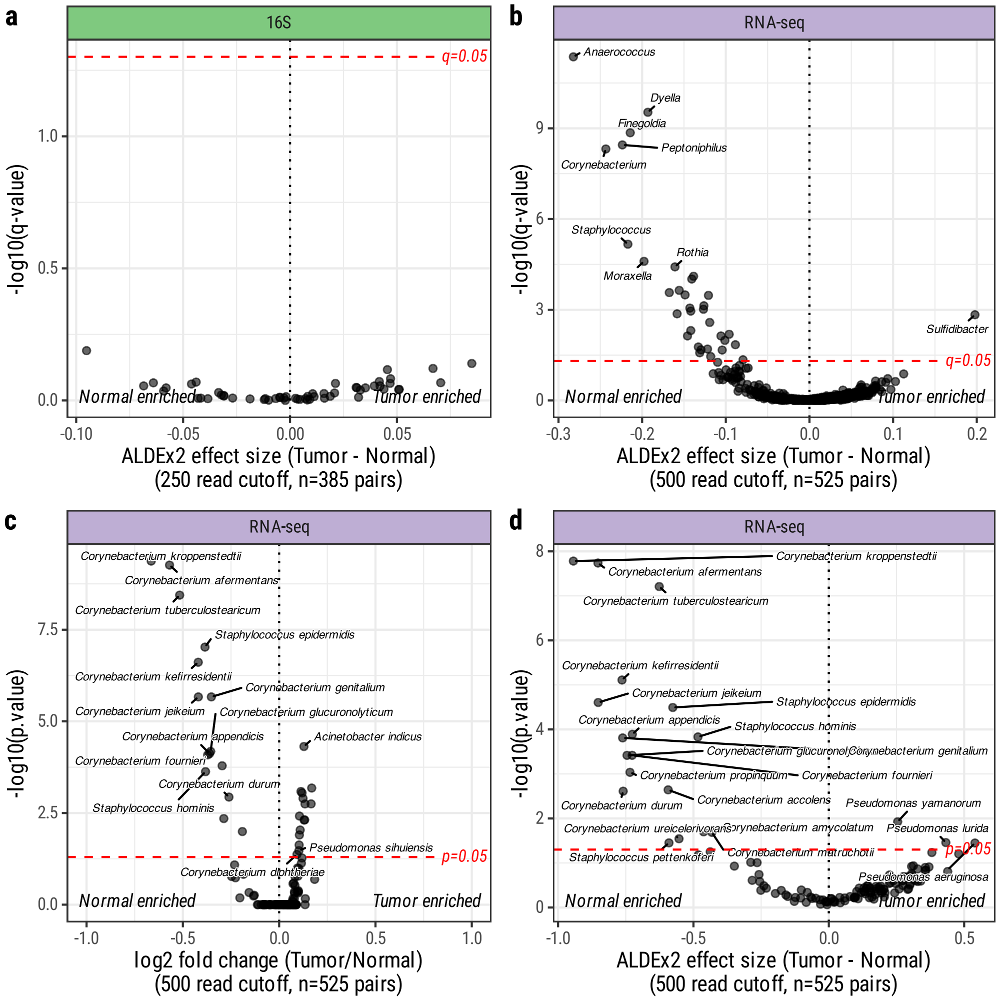

Supplementary Figure 5: Differential abundance analysis at the genus level using ALDEx2 in a) 16S data and b) RNA-seq data. Species-level differential abundance analysis between paired tumor and normal lung tissue RNA-seq samples using c) ANCOM-BC and d) ALDEx2, restricted to species within the most abundant genera (*Acinetobacter*, *Corynebacterium*, *Pseudomonas*, *Staphylococcus*, and *Streptococcus*).

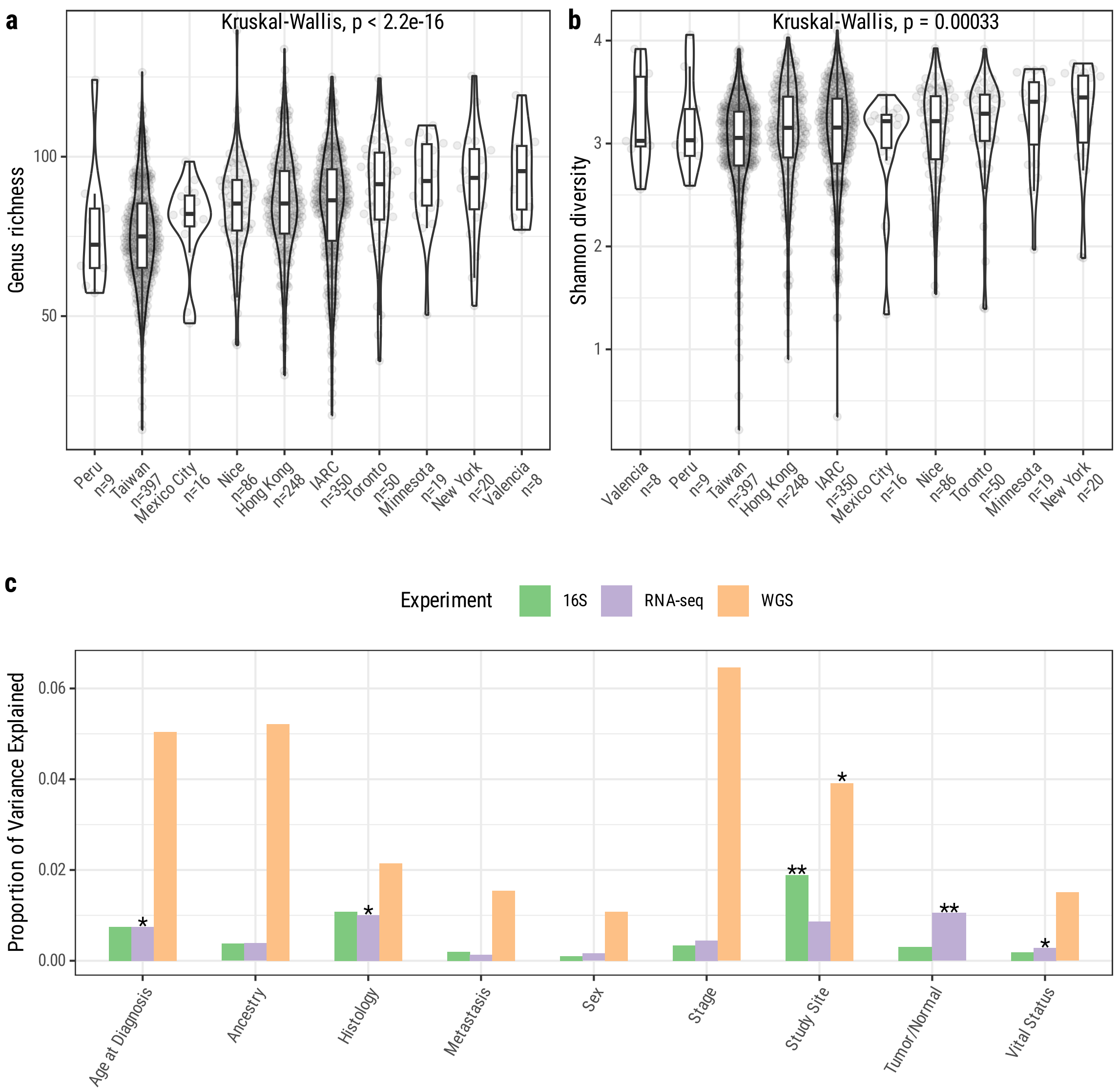

Supplementary Figure 6: a) Comparison of total bacterial richness and b) transcriptomic alpha diversity between different hospital sites using RNA-seq. c) Proportion of beta diversity variance attributed to clinical variables across all three datasets, based on Bray-Curtis dissimilarity. RNA-seq (*n*=803), 16S (*n*=606), and WGS (*n*=369) samples were rarefied to 500, 250, and 100 reads, respectively. *FDR<=0.05; **FDR<=0.01.

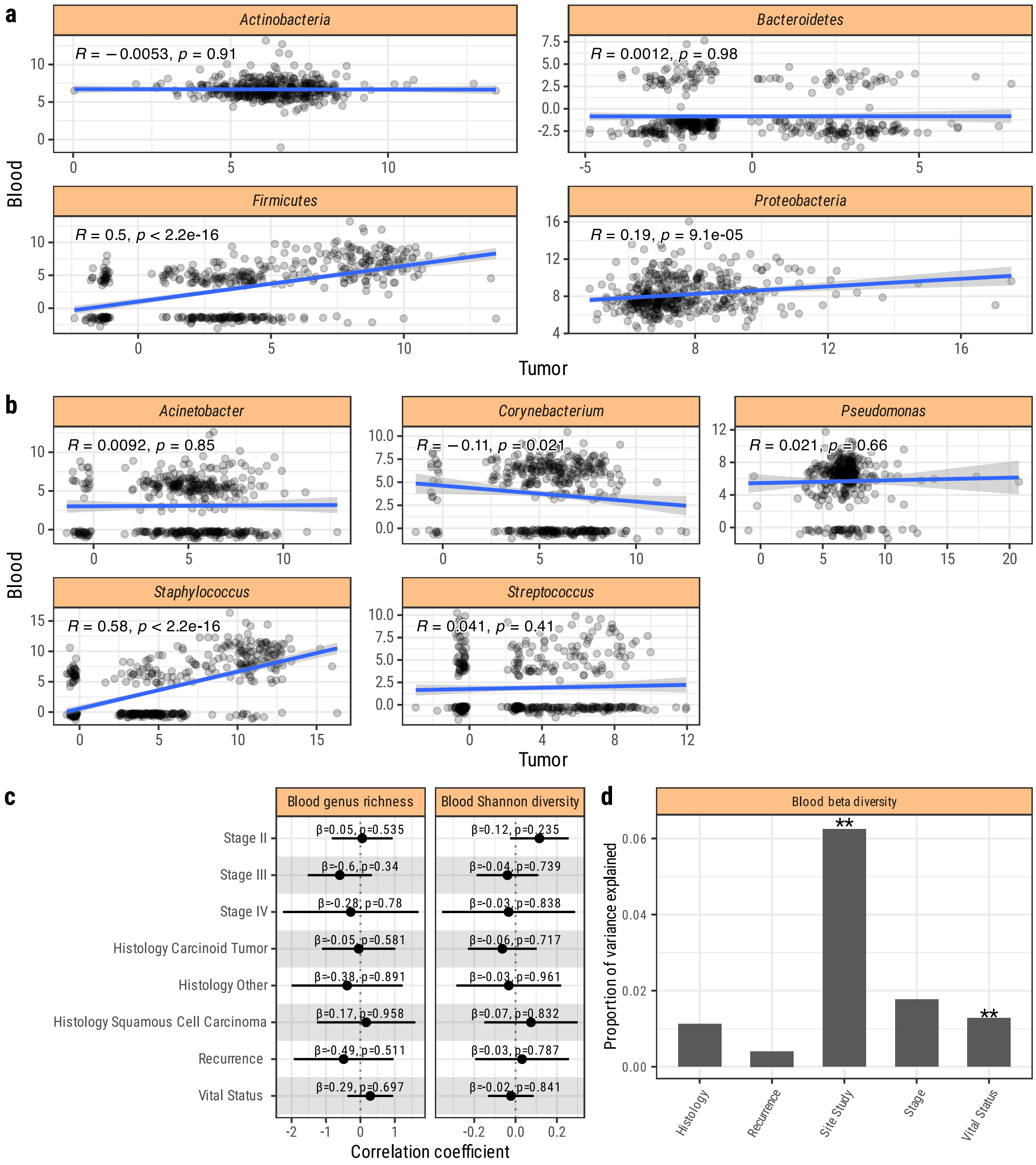

Supplementary Figure 7: Association between bacterial reads in WGS blood samples and clinical features (n=477). Correlation of a) phylum-level and b) genus-level CLR-transformed abundances between tumors and paired blood samples, filtered to the most prevalent phyla and genera. c) Associations between genus richness and Shannon alpha diversity, at rarefaction depth of 100 reads, versus tumor clinical variables using a generalized linear model, adjusted for study site. Error bars signify standard error. Stage I tumors and adenocarcinoma histology serve as references. d) Proportion of beta diversity variance in WGS blood explained by study site and clinical variables. All associations are not significant after multiple testing correction. **unadjusted *p* <=0.01, ***unadjusted *p*<=0.005.

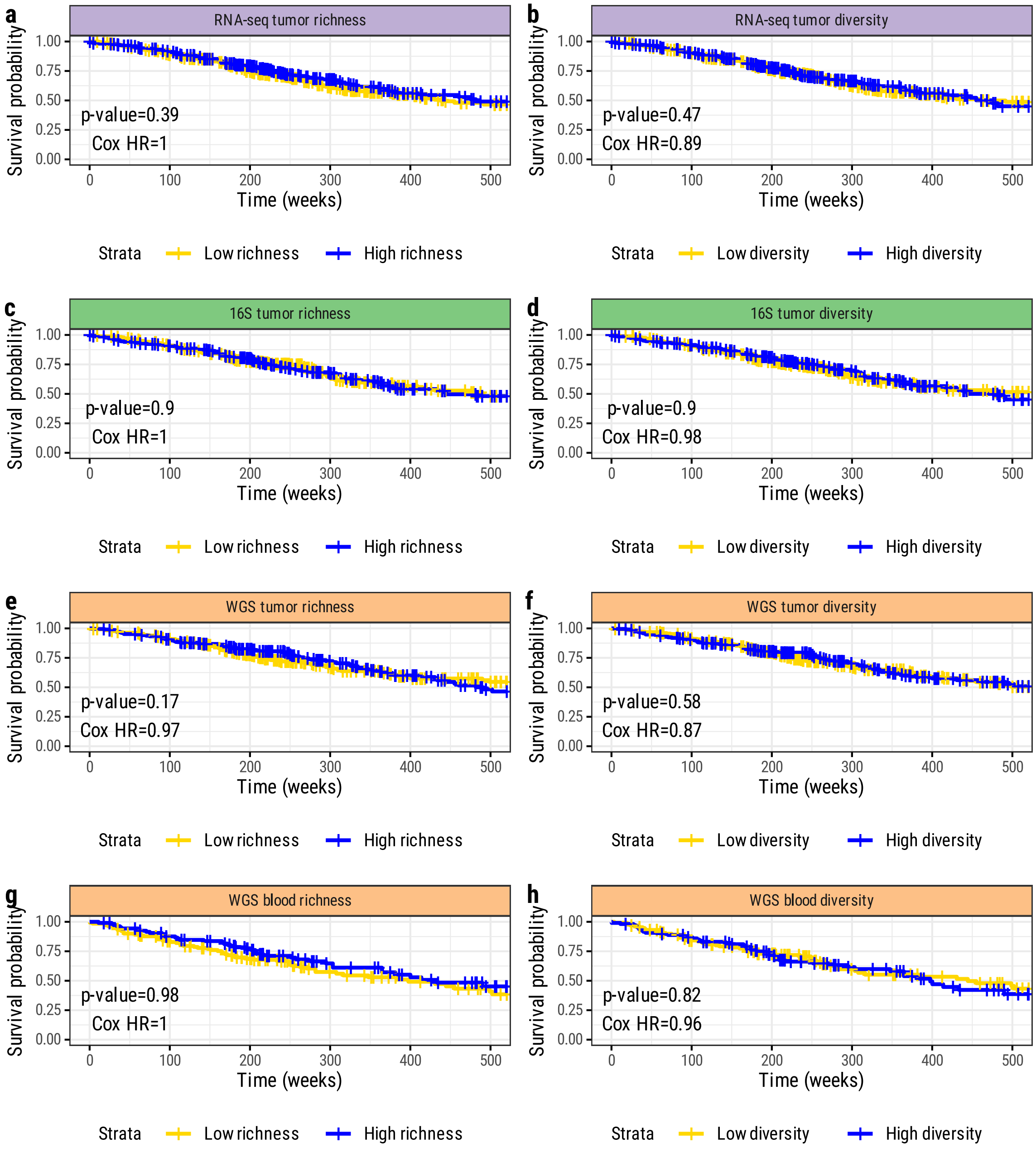

Supplementary Figure 8: a) Overall survival association with RNA-seq tumor genus-level richness and b) Shannon alpha diversity (*n*=587 subjects), c) 16S tumor genus richness and d) Shannon alpha diversity (*n*=488 subjects), e) WGS tumor richness and f) Shannon alpha diversity (*n*=647 subjects), and g) WGS blood genus richness and h) Shannon alpha diversity (*n*=375 subjects). Cox models used continuous values for richness and diversity, which were then partitioned above or below the median within each study site for plotting. RNA-seq, 16S, and WGS samples were rarefied to 500, 250, and 100 genus-level reads, respectively, for diversity and richness calculations.

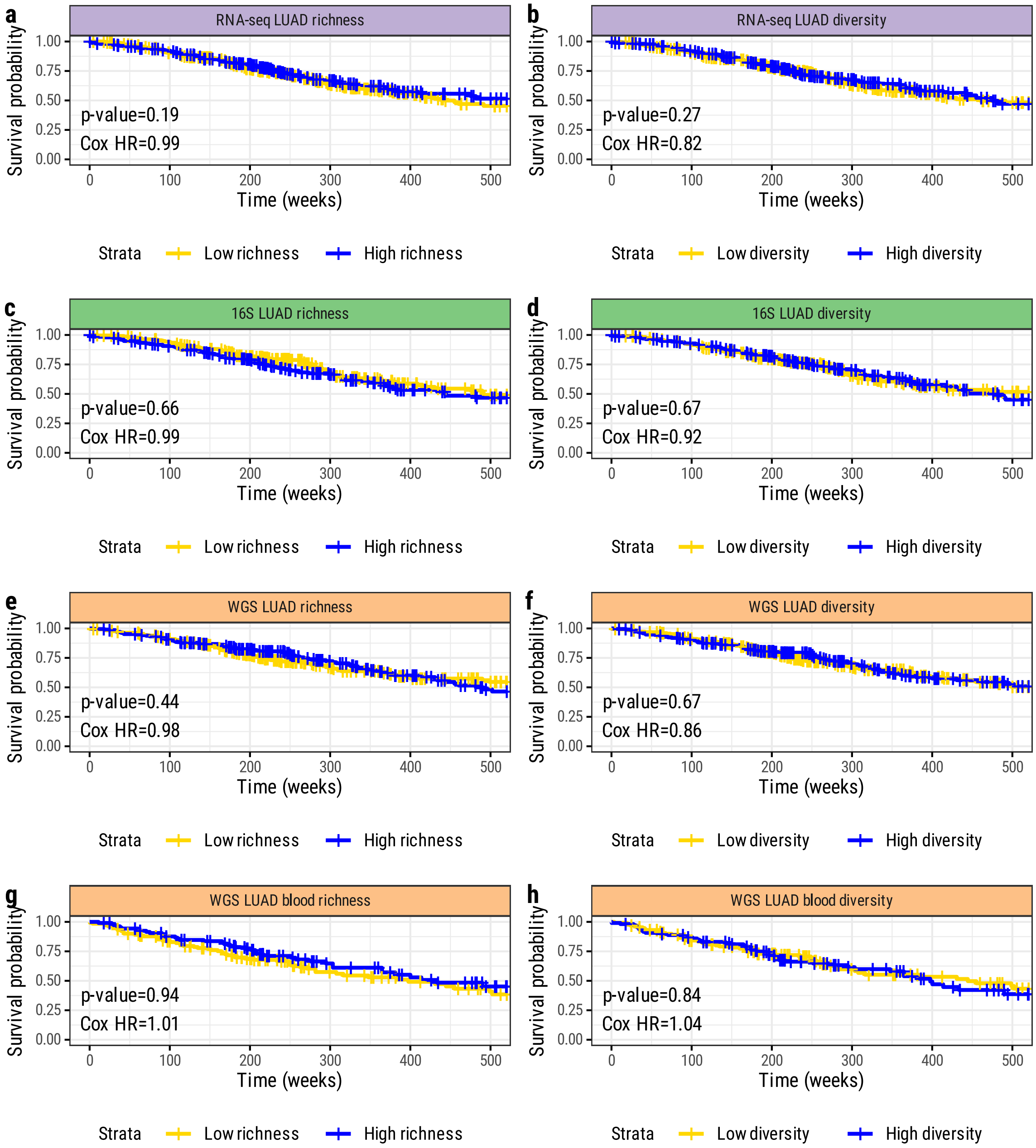

Supplementary Figure 9: Using only patients with lung adenocarcinomas, a) overall survival association with RNA-seq tumor genus-level richness and b) Shannon alpha diversity (*n*=527 subjects), c) 16S tumor genus richness and d) Shannon alpha diversity (*n*=423 subjects), e) WGS tumor richness and f) Shannon alpha diversity (*n*=558 subjects), and g) WGS blood genus richness and h) Shannon alpha diversity (*n*=292 subjects). Cox models used continuous values for richness and diversity, which were then partitioned above or below the median within each study site for plotting. RNA-seq, 16S, and WGS samples were rarefied to 500, 250, and 100 genus-level reads, respectively, for diversity and richness calculations.

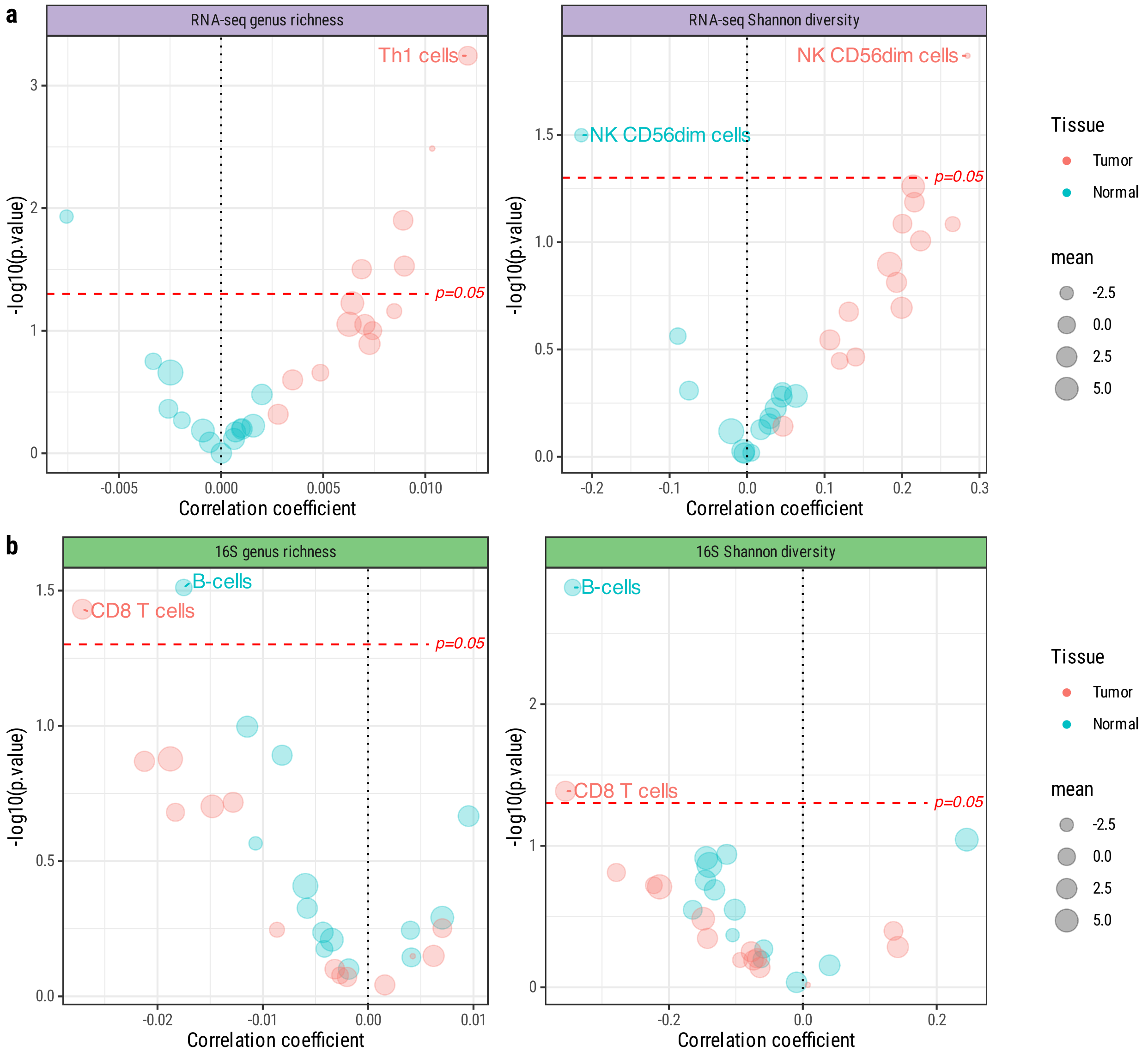

Supplementary Figure 10: a) Correlation of immune cell scores with RNA-seq genus richness and Shannon alpha diversity (*n*=520), and b) 16S genus richness and Shannon alpha diversity (*n*=334 subjects). Only tumor-normal pairs were used for these correlations, adjusted for study site. Point size is scaled according to mean immune score per tissue type. RNA-seq and 16S samples were rarefied to 500 and 250 genus-level reads, respectively, for diversity and richness calculations.

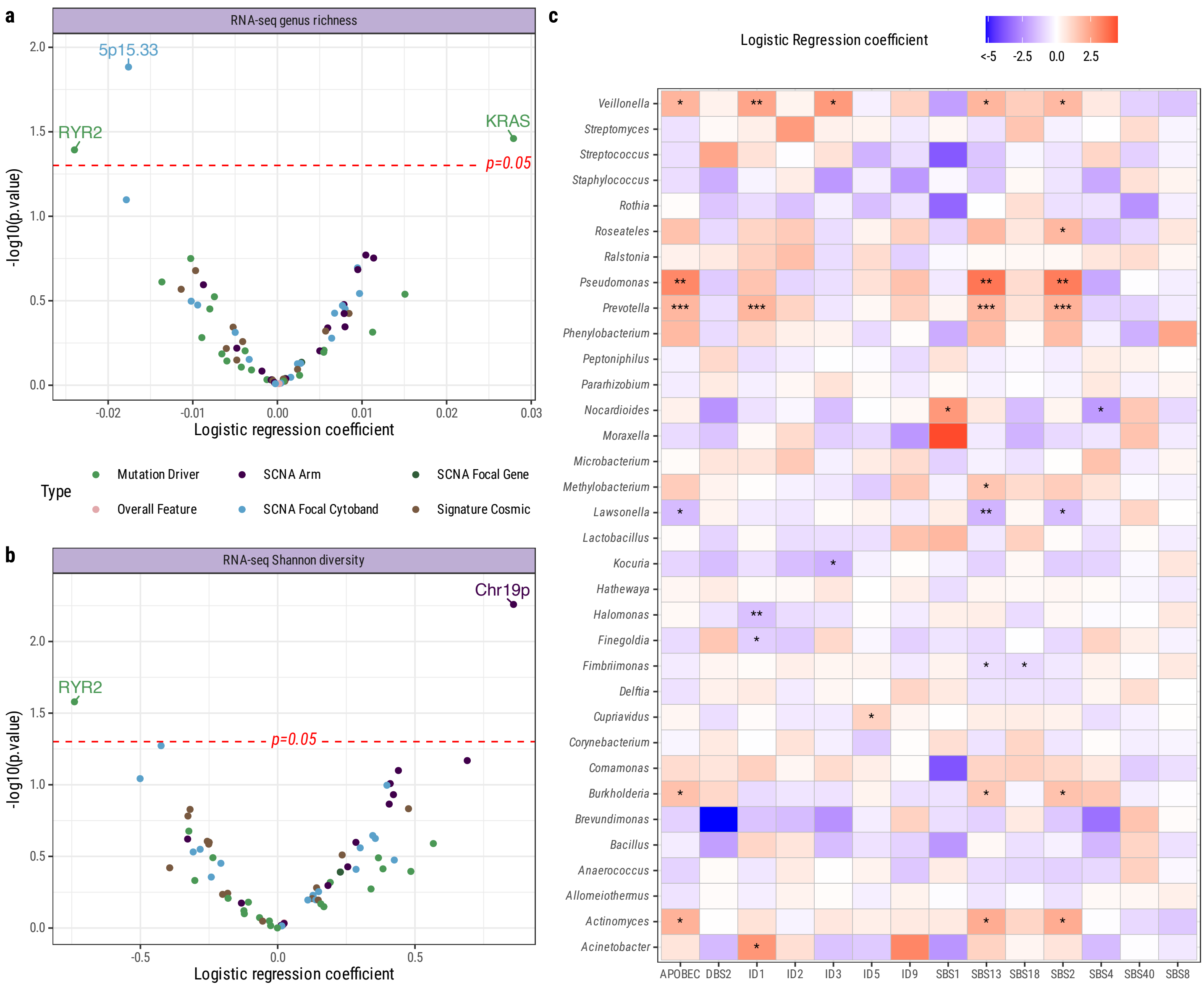

Supplementary Figure 11: Associations between tumor somatic mutations and a) bacterial transcriptomic richness, and b) bacterial transcriptomic Shannon alpha diversity in RNA-seq using binomial regression, adjusted for study site (*n*=346 subjects). Microbiome richness and diversity rarefied to 500 genus-level reads are presented. c) Binomial regression coefficients between center log ratio-transformed (CLR) bacterial relative abundances and genomic mutational signature status (present or absent), adjusted for study site and age at diagnosis (*n*=346 subjects). Only bacterial genera with 50 reads in at least 10% of samples and mutational signatures present in at least 20 tumors were included in this analysis. All associations are not significant (FDR>0.05) after multiple testing correction. *unadjusted $p < 0.05$, **unadjusted $p < 0.01$; ***$0.0001 <$ unadjusted $p < 0.001$.

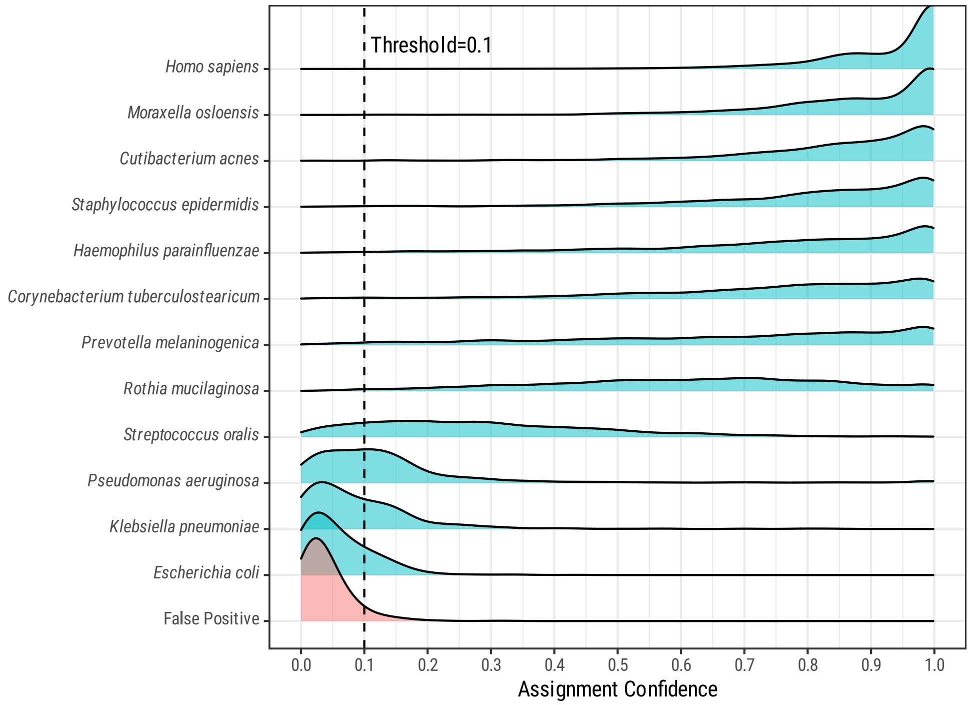

Supplementary Figure 12: Distribution of read assignment confidence with Kraken2. Sequencing reads were simulated for ten bacterial species (in blue) associated with human microbiomes in a 1:100 ratio with sequences simulated from the human genome. False positives are reads assigned to bacterial species other than the ten simulated bacteria.

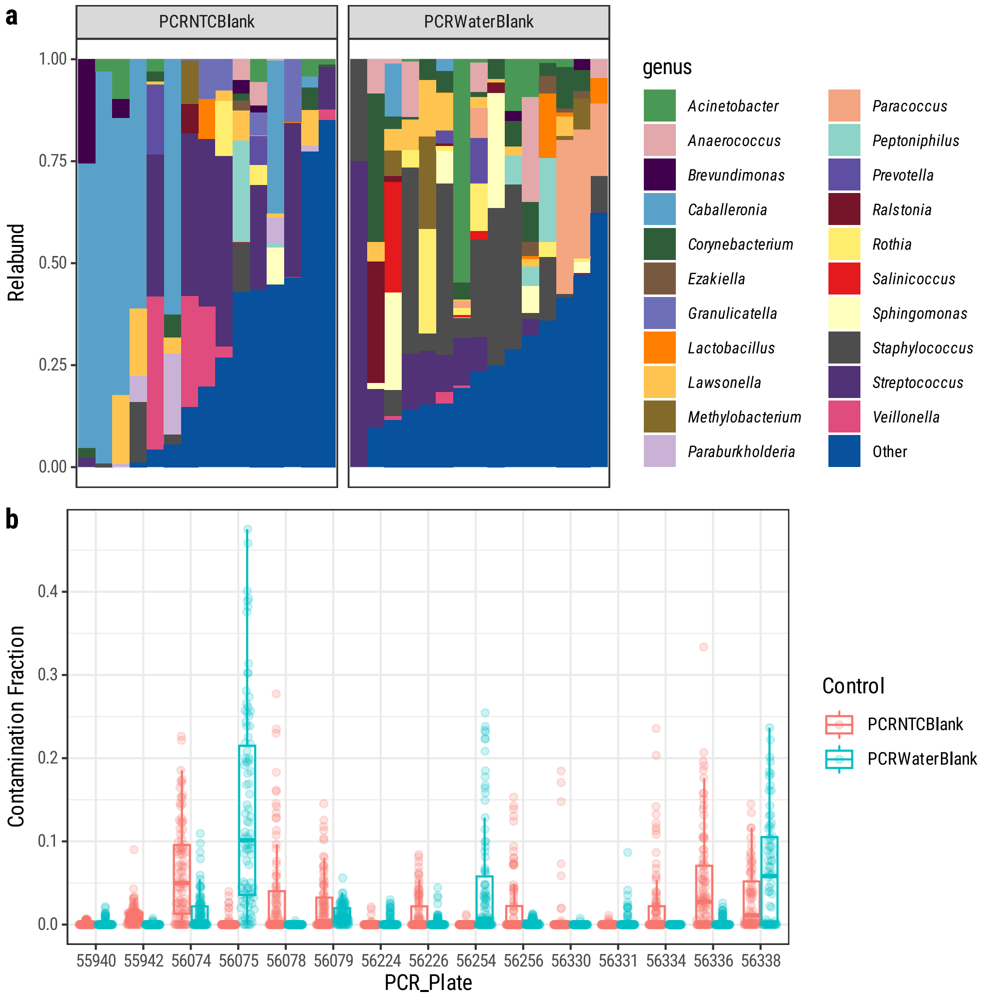

Supplementary Figure 13: a) Genus-level composition of 16S negative controls, b) 16S contamination fractions identified by SCRuB per PCR batch and control type. PCRNTCBlank=PCR no template negative control, PCRWaterBlank=PCR water negative control.
