# Supplementary material for: Microbiome analysis of 940 lung cancers in never-smokers reveals lack of clinically relevant associations
Source: Nat Commun. 2025 Dec 12;17:192. doi: 10.1038/s41467-025-66780-y (PMC12780107; doi:10.1038/s41467-025-66780-y)
Supplement: Supplementary file 2 — Description of Additional Supplementary Files [file 41467_2025_66780_MOESM2_ESM.pdf]

File Name: Supplementary Data 1
Description: Samples and metadata

File Name: Supplementary Data 2
Description: 16S reads matrix, read values of 0 removed

File Name: Supplementary Data 3
Description: WGS reads matrix, read values of 0 removed

File Name: Supplementary Data 4
Description: RNA-seq reads matrix, read values of 0 removed

File Name: Supplementary Data 5
Description: Beta-diversity intra-class correlation associations between lung and tumor tissues sequenced using two or more of 16S, WGS, and RNA-seq, sampling depth at 250 reads. P-values are unadjusted for multiple testing.

File Name: Supplementary Data 6
Description: Differential abundance results at the genus level using 16S data, including both ANCOM and ALDEx2 results

File Name: Supplementary Data 7
Description: Differential abundance results at the genus level using RNA data, including both ANCOM and ALDEx2 results

File Name: Supplementary Data 8
Description: Differential abundance results at the species level using RNA data, including both ANCOM and ALDEx2 results

File Name: Supplementary Data 9
Description: Genomic features associated against microbiome features

File Name: Supplementary Data 10
Description: Literature-based contaminant list
