# Supplementary material for: Microbiome analysis of 940 lung cancers in never-smokers reveals lack of clinically relevant associations
Source: Nat Commun. 2025 Dec 12;17:192. doi: 10.1038/s41467-025-66780-y (PMC12780107; doi:10.1038/s41467-025-66780-y)
Supplement: Supplementary file 4 — Reporting Summary [file 41467_2025_66780_MOESM4_ESM.pdf]

Corresponding author(s): Maria Teresa Landi

Last updated by author(s): YYYY-MM-DD

Reporting Summary

Nature Portfolio wishes to improve the reproducibility of the work that we publish. This form provides structure for consistency and transparency in reporting. For further information on Nature Portfolio policies, see our Editorial Policies and the Editorial Policy Checklist.

Statistics

For all statistical analyses, confirm that the following items are present in the figure legend, table legend, main text, or Methods section.

n/a	Confirmed	
☐	☒	The exact sample size (*n*) for each experimental group/condition, given as a discrete number and unit of measurement
☐	☒	A statement on whether measurements were taken from distinct samples or whether the same sample was measured repeatedly
☐	☒	The statistical test(s) used AND whether they are one- or two-sided<br>*Only common tests should be described solely by name; describe more complex techniques in the Methods section.*
☐	☒	A description of all covariates tested
☐	☒	A description of any assumptions or corrections, such as tests of normality and adjustment for multiple comparisons
☐	☒	A full description of the statistical parameters including central tendency (e.g. means) or other basic estimates (e.g. regression coefficient) AND variation (e.g. standard deviation) or associated estimates of uncertainty (e.g. confidence intervals)
☐	☒	For null hypothesis testing, the test statistic (e.g. *F*, *t*, *r*) with confidence intervals, effect sizes, degrees of freedom and *P* value noted<br>*Give P values as exact values whenever suitable.*
☒	☐	For Bayesian analysis, information on the choice of priors and Markov chain Monte Carlo settings
☒	☐	For hierarchical and complex designs, identification of the appropriate level for tests and full reporting of outcomes
☐	☒	Estimates of effect sizes (e.g. Cohen's *d*, Pearson's *r*), indicating how they were calculated

Our web collection on statistics for biologists contains articles on many of the points above.

Software and code

Policy information about availability of computer code

Data collection	No software was used
Data analysis	Kraken2, https://github.com/DerrickWood/kraken2;Bracken, https://github.com/jenniferlu717/Bracken;Trimmomatic https://github.com/timflutre/trimmomatic; R vegan, https://cran.r-project.org/web/packages/vegan/index.html;ALDEx2, https://www.bioconductor.org/packages/release/bioc/html/ALDEx2.html; ANCOM-BC, https://github.com/FrederickHuangLin/ANCOMBC; SCRuB, https://github.com/Shenhav-and-Korem-labs/SCRuB;InSilicoSeq, https://github.com/HadrienG/InSilicoSeq; analytic code desposited on GitHub, https://github.com/jpmcelderry/Sherlock-microbiome.

For manuscripts utilizing custom algorithms or software that are central to the research but not yet described in published literature, software must be made available to editors and reviewers. We strongly encourage code deposition in a community repository (e.g. GitHub). See the Nature Portfolio guidelines for submitting code & software for further information.

Data

Policy information about availability of data

All manuscripts must include a data availability statement. This statement should provide the following information, where applicable:
- Accession codes, unique identifiers, or web links for publicly available datasets
- A description of any restrictions on data availability
- For clinical datasets or third party data, please ensure that the statement adheres to our policy

All microbiome data can be found in the following data archives: whole genome sequencing data, accession phs001697.v2.p1; 16S rRNA gene sequencing data,

BioProject accession PRJNA1337178; RNA-seq data, accession phs003955.v1.p1.

Research involving human participants, their data, or biological material

Policy information about studies with human participants or human data. See also policy information about sex, gender (identity/presentation), and sexual orientation and race, ethnicity and racism.

Reporting on sex and gender	We collected sex information determined based on self-reporting. We used WGS data to validate sex information where available. We compared alpha and beta diversity of samples based on sex. We conducted no further analyses using sex information because we had no a priori hypotheses that sex influences the association between the lung microbiome and clinical outcomes.
Reporting on race, ethnicity, or other socially relevant groupings	We collected ancestry information based on self-reporting, also including data on geographical locations of the individuals. We used WGS data to validate ancestry information where available. We compared alpha and beta diversity of samples based on ancestry, and performed no other analyses based on ancestry.
Population characteristics	We collected the following population characteristics in this study, including: sex, age at diagnosis, tumor stage, histology, and grade, survival status, overall survival, tumor recurrence, previous non-cancer lung diagnoses, history of asthma, second-hand tobacco smoking. Analyses were adjusted for study site, age at diagnosis, tumor histology and stage where appropriate.
Recruitment	We collected bio-specimens from all lung cancer in never-smokers for the Sherlock-Lung study.
Ethics oversight	The NCI exclusively received de-identified samples and data from collaborating centers, had no direct interaction with study subjects, and did not use or generate any identifiable private information, therefore the Sherlock-Lung study was classified as “Not Human Subject Research (NHSR)” according to the Federal Common Rule (45 CFR 46; eCFR.gov). Some tissue specimens were obtained from the IUCPQ Tissue Bank, site of the Quebec Respiratory Health Network Biobank or the FQRS (www.tissuebank.ca) in compliance with Institutional Review Board-approved management modalities. Some samples and data from patients included in this study were provided by the INCLIVA Biobank (PT17/0015/0049), integrated in the Spanish National Biobanks Network and in the Valencian Biobanking Network, and they were processed following standard operating procedures with the appropriate approval of the Ethics and Scientific Committees. All collaborating centers obtained informed consent for publication of human data from participants under protocols approved by their respective Institutional Review Boards (IRBs).

Note that full information on the approval of the study protocol must also be provided in the manuscript.

Field-specific reporting

Please select the one below that is the best fit for your research. If you are not sure, read the appropriate sections before making your selection.

☒ Life sciences ☐ Behavioural & social sciences ☐ Ecological, evolutionary & environmental sciences

For a reference copy of the document with all sections, see nature.com/documents/nr-reporting-summary-flat.pdf

Life sciences study design

All studies must disclose on these points even when the disclosure is negative.

Sample size	RNA-seq (n=1,203 samples), 16S rRNA gene sequencing (n=1,264 samples), and whole-genome sequencing data (n=1,623 samples) from tumor tissue, adjacent normal lung tissue, and peripheral blood was collected from a total of 940 never-smokers with primary lung cancer, as determined by expert lung cancer pathologists. Power calculations were included to verify statistical power for differential abundance and survival analyses.
Data exclusions	Whole-genome sequencing data was excluded from diversity comparisons because the normal lung tissue was sequenced at lower depth compared to tumors (34x human genome sequencing depth in normal tissue compared to 87x in tumor tissue) and contained too few bacterial reads for robust comparison. Samples with exceedingly low read depth after decontamination (defined as <500 reads in RNA-seq, <250 reads in 16S, and <100 reads in WGS) were excluded from all clinical and demographic associations. Patients without both paired normal and tumor tissues within the same dataset were excluded from all tumor-normal comparisons.
Replication	We replicated all findings using two or more sequencing modalities.
Randomization	For the experiments and analyses performed in this study, randomization is not needed.
Blinding	Data collection only included lung cancer, so blinding was not appropriate.

Reporting for specific materials, systems and methods

We require information from authors about some types of materials, experimental systems and methods used in many studies. Here, indicate whether each material, system or method listed is relevant to your study. If you are not sure if a list item applies to your research, read the appropriate section before selecting a response.

Materials & experimental systems

n/a	Involved in the study
☒	☐ Antibodies
☒	☐ Eukaryotic cell lines
☒	☐ Palaeontology and archaeology
☒	☐ Animals and other organisms
☒	☐ Clinical data
☒	☐ Dual use research of concern
☒	☐ Plants

Methods

n/a	Involved in the study
☒	☐ ChIP-seq
☒	☐ Flow cytometry
☒	☐ MRI-based neuroimaging

Plants

Seed stocks

Report on the source of all seed stocks or other plant material used. If applicable, state the seed stock centre and catalogue number. If plant specimens were collected from the field, describe the collection location, date and sampling procedures.

Novel plant genotypes

Describe the methods by which all novel plant genotypes were produced. This includes those generated by transgenic approaches, gene editing, chemical/radiation-based mutagenesis and hybridization. For transgenic lines, describe the transformation method, the number of independent lines analyzed and the generation upon which experiments were performed. For gene-edited lines, describe the editor used, the endogenous sequence targeted for editing, the targeting guide RNA sequence (if applicable) and how the editor was applied.

Authentication

Describe any authentication procedures for each seed stock used or novel genotype generated. Describe any experiments used to assess the effect of a mutation and, where applicable, how potential secondary effects (e.g. second site T-DNA insertions, mosiacism, off-target gene editing) were examined.
